# Accuracy of digital photographs for assessing inflammatory gum disease in epidemiologic studies

**DOI:** 10.3389/froh.2025.1667604

**Published:** 2025-09-02

**Authors:** Paul D. Terry, O. Lee Wilson, Matthew L. Heaton, Orpheus Triplett, R. Eric Heidel, Rajiv Dhand

**Affiliations:** 1 Department of Medicine, University of Tennessee Health Science Center College of Medicine, Knoxville, TN, United States; 2 Department of General Dentistry, University of Tennessee Health Science Center College of Medicine, Knoxville, TN, United States; 3 Knoxville Periodontics, Knoxville, TN, United States; 4 College of Dentistry, University of Tennessee Health Science Center, Memphis, TN, United States; 5 Department of Surgery, Graduate School of Medicine, University of Tennessee Health Science Center College of Medicine, Knoxville, TN, United States

**Keywords:** epidemiologic studies, inflammatory gum disease, dental health, chronic diseases, remote assessment

## Abstract

**Background:**

Incorporating gum disease assessment into epidemiologic studies would facilitate investigations of disease etiology.

**Objective:**

We evaluated the accuracy and inter-rater reliability of experienced dental health professionals' visual assessments of digital photographs to determine inflammatory gum disease.

**Methods:**

Raters viewed anonymized photographs of the teeth and gums of 30 adult patients and were asked to distinguish “healthy” gingiva from “gum disease” and to assess disease severity. Frequency, percentage, and cross-tabulation statistics were used to perform diagnostic calculations including sensitivity, specificity, and overall accuracy. Fleiss' Kappa, with a 95% confidence interval, was used to test for interrater reliability amongst the four raters. Cohen's Kappa was then calculated for each potential pairing of the four raters.

**Results:**

The accuracy of determining active inflammatory gum disease from digital photographs ranged from 76.7% to 96.7% (mean 85.9%) across the four raters. Sensitivity ranged from 70% to 95% (mean 82.5%), and specificity ranged from 80% to 100% (mean 92.5%). However, inter-rater reliability for disease severity was only fair, with Fleiss's Kappa for gingivitis and periodontitis 0.25 (0.00–0.51) and 0.28 (0.03–0.54), respectively.

**Conclusion:**

Our findings show that digital photographs could be useful for assessing inflammatory gum disease in epidemiologic studies of inflammation-mediated chronic systemic diseases.

## Introduction

Gingivitis is gum inflammation caused by bacterial plaque. Signs of gingivitis include red, swollen gums that can easily bleed, for example, when brushing (1). This early-stage inflammatory gum disease can progress to periodontitis, where plaque below the gum causes the inner layer of the gum and bone to pull away from the teeth, often resulting in bone and tooth loss (2). Inflammatory gum disease remains a major public health concern in the U.S., with little overall improvement in the past 20 years. According to the National Institute of Dental and Craniofacial Research, 42% of adults are currently affected by periodontal disease (3). The prevalence of gingivitis is even higher, with most adults affected to varying degrees (4).

Inflammatory gum disease increases systemic inflammation and the risk of several chronic diseases (5). For example, adults with periodontitis have a higher risk of cardiovascular disease (6, 7). Periodontitis, and the systemic inflammation associated with it, also appear to promote diabetes, which, in turn, can worsen periodontitis in what has been theorized to be an inflammatory “vicious cycle.” (7, 8) Evidence of a vicious cycle includes a three-fold increased risk of periodontal disease in diabetics (8, 9) and improved glycemic control after periodontal treatment (10, 11). Periodontal disease is associated with rheumatoid arthritis occurrence and severity (12), Alzheimer's disease (13), and may contribute to cancer development and growth (14), for example, through an impaired immune surveillance system (15). Although less evidence exists for gingivitis alone, this inflammatory condition of the gingival tissue experimentally increased measures of systemic inflammation (16). Therefore, epidemiologic studies of chronic disease etiology, treatment and/or prevention may increasingly seek to incorporate measures of dental health into risk factor assessments and data analyses.

Epidemiologic studies focused on gum disease traditionally rely on direct clinical examination (17), which can be prohibitively expensive and burdensome in large-scale population-based studies, especially when gum disease is not the primary focus of the research. Therefore, assessment of dental health via digital photographs may have considerable advantages for large-scale epidemiology studies. Because the utility of digital photographs to assess inflammatory gum disease in epidemiologic studies remains unclear, we evaluated the accuracy and inter-rater reliability of visual assessment of digital photographs by experienced dentists to determine inflammatory gum disease status in a group of anonymized adult patients.

## Methods

**Figure 1:**
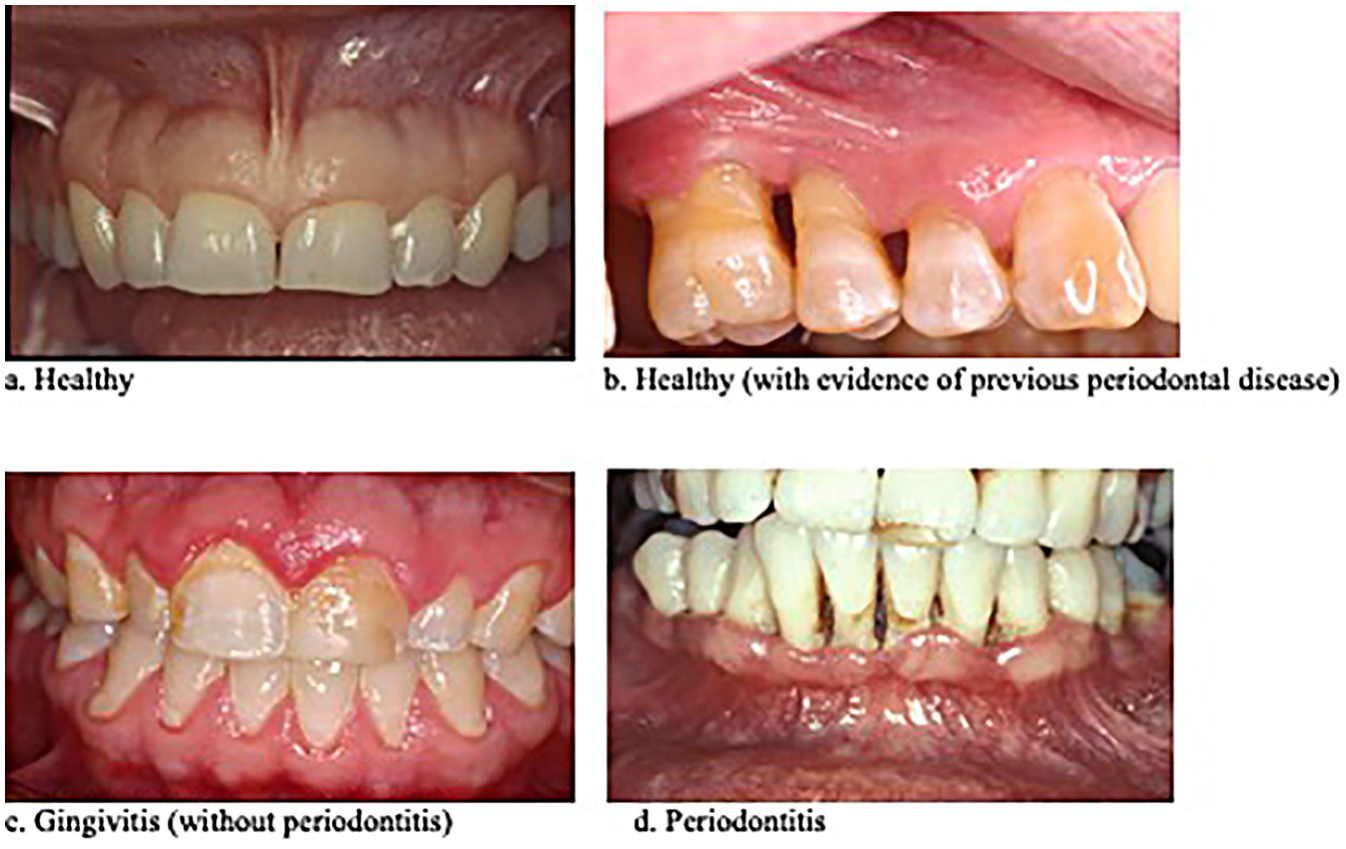
Four categories of photograph used in the study based on the patient's clinical diagnosis.

The research was undertaken at the University of Tennessee Medical Center (UTMC) in Knoxville, Tennessee, and was approved by the UT Graduate School of Medicine's Institutional Review Board. Anonymized digital photographs of the teeth and gums of 30 adult patients were provided by a dental practice in Knoxville, Tennessee, that was not affiliated with the UTMC Department of General Dentistry. Photos of patients were taken during the course of normal clinical care by a local periodontist with decades of clinical experience. Diagnoses were based on clinical examinations and radiographic techniques indicating key distinctions between healthy gums, gingivitis, and more advanced periodontitis. At the time the photographs were taken, each patient was diagnosed clinically and radiographically as having either no current gum disease (*n* = 10), gingivitis only (*n* = 10), or periodontitis (*n* = 10) (Figure 1).

Patients' underlying diagnoses were blinded and the photographs were displayed in random order for four dental health professionals, a periodontist and two dentists at UTMC-Knoxville, and a third dentist at UTHSC College of Dentistry in Memphis, TN. Each evalutor had ties to both community-based and academic dentistry and had decades of experience diagnosing and treating patients with various forms and stages of inflammatory gum disease. Although each assessment relied primarily on the extensive clinical experience and training of the viewing dentist, gingival redness, edema, flattening of papillae, gum recession, and signs of periodontal bone loss, were considered when assessing the photographs. The photographs were viewed by each rater separately to help ensure independent assessments. In the first round, raters were asked to distinguish between currently “healthy” and “inflamed” gum tissue. Then, considering only the digital photographs of patients diagnosed with inflammatory gum disease, raters were asked to further distinguish between “gingivitis only” and “periodontitis.” Because some of the “healthy” patients had previous gum disease that was in remission, the raters were asked to distinguish “healthy” (Figure 1A) from “currently healthy with evidence of previous periodontal disease” (Figure 1B). The latter category was deemed important because of the high risk of periodontitis relapse in those individuals, which would be a consideration in epidemiologic studies.

To obtain previous studies that assessed gum disease from digital photographs, searches were conducted of the PubMed database using search-terms such as “oral disease,” “gum disease,” “gingivitis,” “periodontal disease,” “periodontitis,” “digital photographs,” and by cross-referencing citations in identified studies that were available in print or online before July 1, 2025. Although some of the retained studies included adolescents in their study populations, we did not consider studies that focused primarily on children. It was not our aim to conduct a full systematic review due to the lack of previous studies that relied on visual assessment by trained dental care providers.

Frequency, percentage, and cross-tabulation statistics were used to perform diagnostic calculations including sensitivity, specificity, and overall accuracy. Fleiss' Kappa with a 95% confidence interval was used to test for interrater reliability amongst the four raters. Cohen's Kappa was then calculated for each potential pairing of the four raters. Statistical significance was assumed at an alpha value of 0.05 and all analyses were performed using SPSS Version 29 (Armonk, NY: IBM Corp.).

## Results

The accuracy of determining active inflammatory gum disease (gingivitis and/or periodontitis) from digital photographs ranged from 76.7% to 96.7% (mean = 85.9%) across the four raters (Table 1). Sensitivity ranged from 70% to 95% (mean = 82.5%), and specificity ranged from 80% to 100% (mean = 92.5%). Approximately half of the currently “healthy” patients showed signs of periodontal disease in remission. In two of these cases, a rater incorrectly diagnosed active gum disease (data not shown).

**Table 1:** Sensitivity and specificity of visual assessment of gum disease from digital photographs: “healthy” vs. “gum disease” (gingivitis and/or periodontitis).

Rater	Healthy	Gum disease	Sensitivity	Specificity	Accuracy
Rater 1	10/10	19/20	95% (76%−99%)	100%	96.7% (83%−99%)
Rater 2	10/10	15/20	75% (53%−89%)	100%	83.3% (66%−93%)
Rater 3	9/10	14/20	70% (48%−85%)	90% (60%−98%)	76.7% (59%−88%)
Rater 4	8/10	18/20	90% (70%−97%)	80% (49%−94%)	86.7% (70%−95%)

When considering only patients with inflammatory gum disease, inter-rater reliability for disease severity was only fair (Table 2), with Fleiss's Kappa for gingivitis and periodontitis 0.25 (0.00–0.51) and 0.28 (0.03–0.54), respectively. The accuracy of distinguishing gingivitis from periodontitis ranged from 50.0% to 66.7% (mean = 62.5%).

**Table 2:** Inter-rater reliability for inflammatory gum disease severity (gingivitis vs. periodontitis).

Gum disease	Fleiss's Kappa (across all raters)	*p*-value	Rater combinations	Cohen's Kappa	*p*-value
Gingivitis	0.25 (95% CI 0.00–0.51)	0.05			
			Rater 1/Rater 2	0.24	0.43
			Rater 1/Rater 3	0.09	0.75
			Rater 1/Rater 4	0.35	0.26
			Rater 2/Rater 3	0.29	0.20
			Rater 2/Rater 4	0.44	0.09
			Rater 3/Rater 4	0.21	0.49
Periodontitis					
	0.28 (95% CI 0.03–0.54)	0.03*			
			Rater 1/Rater 2	0.23	0.43
			Rater 1/Rater 3	0.62*	0.04*
			Rater 1/Rater 4	0.29	0.20
			Rater 2/Rater 3	0.58	0.07
			Rater 2/Rater 4	0.14	0.39
			Rater 3/Rater 4	0.14	0.39

*p < 0.05.

When considering patients currently without inflammatory gum disease, raters identified approximately half of these 10 “healthy” patients as having periodontitis in remission. However, only the patients' diagnoses at the time the photographs were taken were ultimately known, with no “gold standard” diagnosis previous to that time available, so we could not calculate measures of accuracy for distinguishing gum disease in remission. Nonetheless, a rater incorrectly assessed active inflammatory gum disease in two “healthy” patients that the other raters considered “currently healthy with evidence of previous periodontal disease.” The rater's errors in diagnosing active gum disease in currently healthy patients were incorporated into the estimates of accuracy shown in Table 1.

## Discussion

We found that experienced dentists could distinguish healthy from inflamed gum tissue with good accuracy even though individuals with periodontitis in remission were included in the healthy group, reflecting real world applications. As expected, however, the inter-rater reliability regarding “gingivitis only” vs. “periodontitis,” as measured by Fleiss's Kappa, was only fair. The latter result was expected due to the well-recognized inability of digital photographs to expose subtle changes in bone density and structure, which otherwise can be discerned with good accuracy from clinical examination and x-rays. Nonetheless, our data support the utility of distinguishing inflammatory gum disease from healthy gums using digital photographs.

Increasing evidence suggests that inflammatory gum disease can fuel the development, progression, and treatment intransigence, of several common and debilitating chronic diseases, likely through pathways related to systemic inflammation (5–16). Whereas epidemiologic investigations of chronic diseases are likely to assess data on tobacco smoking, for example, and other known or suspected chronic disease risk factors, the assessment of inflammatory gum disease in epidemiologic studies has been rare. There are several reasons for this, including a general lack of awareness of how important gum disease may be in the occurrence, development and treatment efficacy of several chronic diseases, and the logistic and financial burdens of assessing gum disease in large-scale population-based studies. Clinical oral examination and x-rays, the gold standard for assessing gum disease, is consequently rarely done in large scale epidemiologic studies, especially when gum disease is not the primary focus of the research. Examiner fatigue, low patient participation, high dropout rates, and high risk of observer bias, are other problems noted with clinical oral examinations in epidemiologic studies (17). Therefore, assessment of dental health using digital photographs has advantages for large-scale epidemiologic studies, where costs, risk of observer bias, and burdens on study participants and staff, are greatly reduced. Moreover, study participants, as well as people in the general population, are often not aware of the status of their dental health and/or do not report it accurately (18). Given all of these considerations, our study's findings may have implications for the widescale incorporation of gum disease assessment in population-based epidemiologic studies of chronic diseases.

Our literature search yielded 19 previous studies that assessed gum disease using digital photographs (Table 3) (19–37), with most published in the past four to five years. Data from over 5,000 patients were analyzed in these studies from East (*n* = 11) and South (*n* = 3) Asia, the Middle East (*n* = 4), and Europe (*n* = 1). Sample sizes ranged in from *n* = 20 to *n* = 1,333 participants, among whom a minority were children and young adults. Estimates of accuracy in assessing inflammatory gum disease from digital photographs generally ranged from approximately 0.7–0.9 in those studies. One study (25) calculated the sensitivity and specificity of visual assessment of gingivitis (sensitivity = 67.2%, specificity = 85.2%). Of note, the estimates of sensitivity and specificity obtained for the visual assessment (25) regarding gum disease were similar to those obtained for the complex algorithms and computer software (Table 3). As noted earlier, our estimates of accuracy were also consistent with those from AI-based software.

**Table 3:** A selection of studies that assessed inflammatory gum disease from digital photographs.

Author, year, location	Population	Study objective/rationale	Assessment tools, measures	Results	Use in population-based studies	Caveats
Seshan, 2012, India (19)	20 volunteers with gingival inflammation, 15–55 years old	Use digital photos to assess changes in gingival inflammation pre- vs. post-treatment	Serif photo pluse-6 software to assess redness and tooth surface area between inter-proximal papillae and gingival margin	The software detected some statistically significant differences in redness and swelling pre- vs. post-treatment	Pre- vs. post-treatment is different from comparing data from separate individuals	Investigators did not assess signs of bone loss or periodontitis severity
Rana, 2017, India (20)	150 adults, 18–90 years old	Use color-enhanced digital photos and software to detect early periodontitis	Machine/deep learning software that provides gingival inflammation data using special fluorescent light	The software distinguished inflamed from healthy gingiva (area under the curve = 0.75; precision and recall values were 0.347 and 0.621, respectively)	Complex computer models may not be feasible in large population-based studies	Investigators did not assess signs of bone loss or periodontitis severity
Joo, 2019, South Korea (21)	1,109 training photos + 150 for validation	To classify degree of periodontitis with software	A convoluted neural networks model	The model has moderate accuracy for classifying periodontitis (accuracy = 81%)	Complex computer models may not be feasible in large population-based studies	The model had trouble “adjusting” to new data
Moriyama, 2019, Japan (22)	1,333 dental patients	Estimate depth of 12 pockets on the buccal side of 4 upper front teeth	MapReduce-like (deep learning) periodontal pocket depth estimation model	Model showed an accuracy = 76.5%, which was higher with severe disease (accuracy = 91.7%)	Complex computer models may not be feasible in large population-based studies	The novel model requires further validation
Chen, 2020, China (23)	Photos of 90 healthy gums and 90 with gingivitis	To diagnose gingivitis more efficiently and accurately	Gingivitis recognition based on Gray-Level Co-Occurrence Matrix, Artificial Neural Network, and Genetic Algorithms	Model demonstrated higher accuracy than Contrast Limited Adaptive Histogram Equalization and other programs tested (sensitivity =75.1%; specificity = 75.8%; accuracy = 75.9%)	Complex computer models may not be feasible in large population-based studies	Periodontitis status was not assessed
Alalharith, 2020, Saudi Arabia (24)	47 orthodontic patients	Test the developed convoluted neural network models for accuracy in detecting gingivitis	Region-based convoluted neural network models using ResNet-50 convolutional Neural Network	Model showed good accuracy (77.1%)	Complex computer models may not be feasible in large population-based studies	Periodontitis status was not assessed
Liu, 2020, China (25)	35 images from dental clinics	Evaluate several dental conditions, including periodontitis, using AI	A Smart Dental Health-IoT Platform Based on Intelligent Hardware, Deep Learning, and Mobile Terminal	For periodontal disease: Sensitivity = 0.097 Specificity = 0.95	Complex computer models may not be feasible in large population-based studies	Periodontal disease was not defined, with unclear “Gold Standard” used in analyses
Guo, 2021, China (26)	31 healthy college students	Evaluate gingivitis, plaque, and carries from photos vs. clinical scores	Modified gingivitis index, plaque index, and caries status	Moderate correlation of gingivitis assessment of photos vs. clinical signs (sensitivity = 67.2%; specificity = 85.2%)	Caries status assessment using photos may be feasible, perhaps more so than gingivitis	Healthy students are not a typical target population for chronic disease outcomes
Shrivastava, 2021, India (27)	27 patients with gingivitis and 27 periodontitis	Assess gingival inflammation quantitatively	Pre- vs. post-treatment gingival color changes using Photometric CIELab analysis of photos	Significant differences in gingival color were detected	Pre- vs. post-treatment is different from comparing data from separate individuals	Periodontitis status was not assessed
Li, 2021, China (28)	625 dental patients 14–60 years old	Automatically detect gingivitis, calculus and soft deposits	A Multi-Task Learning convoluted neural network model	The software showed some accuracy detecting dental conditions (area under the curve = 87.1%)	Complex computer models may not be feasible in large population-based studies	Periodontitis status was not assessed
Ginesin, 2022, Israel (29)	40 patients with periodontitis	To assess gingival color during periodontal treatment	CIELab color analysis pre- vs. post-treatment	The system detected a reduction in redness during treatment	Requires software, training, and data analysis, which might not be practical	Redness is not a definitive marker of periodontal disease
Kim, 2023, South Korea (30)	25 orthodontic patients 20–37 years old	Assess the association between gingival redness and gingival index	A computer-based algorithm to compare pre- vs. post-treatment gingival index	An association between gingival redness and gingival index was confirmed, and showed difference pre- vs. post-treatment	The algorithm requires further validation in larger studies and has not been applied to periodontitis	Small sample size; young patients; periodontitis not assessed
Kurt-Bayraktar, 2023, Turkey (31)	654 photos from patients 13 years of age or older	To assess an AI-based software for detection of gingival inflammation and other dental problems	Various programs (YOLO, CSPNet, PANet) were used	Accuracy for gingival inflammation was 0.636	Novel program that is not commercially available	Unclear Gold Standard that did not include clinical exams
Liu, 2024, China (32)	673 oral endoscopic images in a test dataset	Segment intraoral photographic images for the detection of gingivitis	Deep learning programs “Oral-Mamba” and “U-Net”	Accuracy for gingivitis = 0.83	Requires software, training, and data analysis, which might not be practical	The programs are sensitive to the quality and direction of light
Wen, 2024, China (33)	826 patients from children to 50 + years old	To test the accuracy of a novel convoluted neural network (CNN) algorithm	A novel CNN-based gingival inflammation grading algorithm	Sensitivity = 0.82 Specificity = 0.69 Accuracy = 0.74	Novel program that is not commercially available	The “Gold Standard” was not entirely clear
Li, 2024, China (34)	134 volunteers ages 14–64 years	To evaluate the advanced CNN models using ensemble learning	Deep CNN models AlexNet, VGG, GoogLeNet, and ResNet	Area under the curve (AUC) values ranged from 0.89–0.94	Software specific training required	The “Gold Standard” was not entirely clear
Alam, 2024, South Asia and Middle East (35)	60 patients seeking dental care	To evaluate the accuracy of Al algorithms in diagnosing periodontal disease	A deep learning AI algorithm	Sensitivity = 0.90 Specificity = 0.84 Accuracy = 0.87	Software not commercially available	Unclear definition of “periodontal disease” and confusing “Gold Standard” because clinical exams were also assigned accuracy scores
Chau, 2025, China (36)	44 older adults in day-care community centers (age 60+)	Test the accuracy of artificial intelligence (AI) to detect gingivitis using digital photos	GumAI, an artificial intelligence program	Sensitivity = 0.93, Specificity = 0.50, Accuracy = 0.85	Feasible with program procurement and training	The “Gold Standard” was unclear, other than a panel of periodontists
Vaughan, 2025, U.K. (37)	35 undergraduate dental students	Test the accuracy of AI to detect gingivitis using digital photos	SmileMate, an artificial intelligence program	Sensitivity = 1.0 Specificity = 0.091	Feasible with program procurement and training	Poor specificity

All except one previous study used software to assess gum disease. Those studies showed reasonable accuracy discerning inflammatory gum disease from digital photographs, for example, using powerful “deep learning” or similar types of software (Table 3). However, the computer algorithms appear to be specific to each study, require development and maintenance by highly skilled personnel, and may be proprietary and expensive to purchase. Our data suggest that experienced dental health professionals can achieve similar accuracy in diagnosing inflammatory gum disease without the use of complex and costly computer algorithms. In our study, raters were more accurate in discerning patients with active inflammatory gum disease than in categorizing disease severity, i.e., gingivitis vs. periodontitis. However, the latter distinction may be less important because both conditions increase measures of systemic inflammation (16, 38).

Our study has four noteworthy limitations, including its sample size. Each of four raters assessed gum disease in 30 digital photographs, which was sufficient to generate estimates of accuracy and inter-rater reliability with moderate precision. Nonetheless, a larger sample size likely will be needed to increase the precision of these estimates in future studies.

Second, the digital photographs we obtained from an unaffiliated dental practice were not taken using a standardized protocol and, hence, were not uniform in image perspective or lighting (Figure 1). Greater accuracy in gum disease diagnosis may result from using a standardized series of photographs for each patient, for example, a frontal photograph showing labial surfaces of anterior teeth; lateral photographs showing buccal surfaces of left and right posterior teeth; a maxillary dentition photograph showing palatal and occlusal surfaces of maxillary dentition; and a mandibular dentition photograph showing lingual and occlusal surfaces of mandibular dentition, using established protocols regarding photography equipment, lighting, and camera angle. This need not be overly burdensome on study staff or resources, however, because study coordinators could be trained by study dentists to follow such data collection protocols at participant enrollment.

Third, the primary aim of our study was to assess the sensitivity, specificity, and accuracy of the remote assessment of inflammatory gum disease from digital photographs by experienced dentists using a common set of criteria. We did not concurrently assess gum disease from digital photographs using computer software, so we can not directly compare our study results with those of an algorithm-based assessment in our study population. However, based on our results and those of the previous studies we reviewed here, there seems to be no clear evidence of greater diagnostic accuracy of algorithm-based assessment over visual assessment. Likewise, no previous study performed a direct comparison of assessment methods. A comparison between algorithm-based assessments of digital photographs with visual assessments by experienced dentists would be a reasonable aim of future studies.

Finally, although we searched two well-known extensive online databases for published literature related to the assessment of dental health via photographs, and cross-referenced citations in the identified studies in search of additional citations, our review was not a systematic review (39). Therefore, it is possible that we did not obtain one or more of the relevant previous studies.

## Conclusion

Incorporating the assessment of inflammatory gum disease into epidemiologic studies would facilitate investigations of chronic disease etiology as well as those to determine the effect of treating gum disease on the course of several chronic systemic diseases, such as diabetes (11, 12). However, an ongoing question with such studies is how to accurately discern the presence of inflammatory gum disease when clinical examinations and x-rays, the gold standard, are not feasible. Several previous studies assessed the accuracy of discerning gum disease in digital photographs using complex computer algorithms. Our study's findings support the utility of a simpler method that yields similar results and could be readily applied in population-based field studies and large-scale epidemiologic investigations.

## Data Availability

The datasets presented in this article are not readily available because No personal information can be shared without permission. Requests to access the datasets should be directed to pterry@utmck.edu.
